# Economic empowerment and various preventive strategies play a role in reducing asymptomatic malaria towards the end of the rainy season

**DOI:** 10.12688/openresafrica.15809.2

**Published:** 2025-10-06

**Authors:** Chibuike Okpala, Ifeoma Umeh, Linda Onyeka Anagu

**Affiliations:** 1 Pharmaceutical Microbiology and Biotechnology, Nnamdi Azikiwe University Faculty of Pharmaceutical Sciences, Awka, Anambra, Nigeria; 2 Clinical Pharmacy and Pharmacy Management, Nnamdi Azikiwe University Faculty of Pharmaceutical Sciences, Awka, Anambra, Nigeria

**Keywords:** Asymptomatic malaria, rainy season, preventive measures, malaria transmission.

## Abstract

**Background:**

Asymptomatic malaria is responsible for persistent malaria transmission. The prevalence of malaria in children under 5 years in Anambra State, Nigeria, is declining. The sustained transmission of malaria threatens to reverse this decline, as indicated by the high severe malaria cases during the rainy transmission season. We ascertained the prevalence of asymptomatic malaria using the malaria Rapid Diagnostic Test (mRDT) and the determinants of asymptomatic malaria.

**Methods:**

A community-based cross-sectional study was conducted at the end of the rainy season in November 2024 among 130 consenting apparently healthy adults aged 18 years and above residing in the Nnewi North Local Government Area of Anambra State using a standardised self-administered questionnaire and a P. falciparum mRDT. The questionnaire sought information on the participants’ sociodemographic and socioeconomic factors, malaria healthcare-seeking behaviour, use of malaria prevention measures, environmental conditions, and perceptions of malaria risk. A finger-prick was used for the mRDT kit. Data were analysed using Stata 17/BE. Binary logistic regression was used to identify the factors associated with asymptomatic malaria.

**Results:**

Of the 130 participants, 26.15% (34/130) were confirmed to be infected with Plasmodium falciparum. There was an overreliance on personal feelings for malaria diagnosis. Covering water containers around a house could be an effective protective measure against asymptomatic malaria. The odds (odds ratio (OR): 0.29/0.27, 95% (CI): 0.07/0.06 – 1.24) of having asymptomatic malaria were lower in those who agreed that ‘the cost of malaria prevention tools, such as Insecticide-treated nets (ITNs), insecticides and mosquito repellents, is reasonable’ compared to those who did not.

**Conclusion:**

The prevalence of asymptomatic malaria among semi-immune adult participants residing in the Nnewi North Local Government Area (LGA) was 26.15%. Having the financial means to access resources for malaria prevention can lead to healthier behaviours and actions that reduce malaria transmission.

## Introduction

Asymptomatic malaria is a significant driver of persistent malaria transmission and can have varying impacts, including the prevention of malaria elimination, re-establishment of malaria in malaria-free countries, outbreaks, and negative health outcomes for the most vulnerable – pregnant women and children. Malaria is preventable and treatable and contributes to health inequalities. Several complementary control strategies are being deployed to reverse the rise in malaria incidence, currently at 60.4 cases per 1000 compared to 58.6 per 1000 in 2022 ^1^. These strategies include vector control, vaccination, improved diagnosis, chemoprevention, and chemotherapy.

Death from severe malaria is still high, with a total of 597,000 deaths ^1^. Mortality is the highest burden in high-impact countries, with Nigeria having the highest burden ^1^. Within Nigeria, the malaria parasite prevalence in children under 5 years of age in Anambra State has continually decreased from 14.2% in 2010 to 10.2% in 2015 and further reduced to 8.8% in 2018 ^2^. Of the 36 states in Nigeria, Anambra has the second-lowest prevalence of malaria. Parasite prevalence is still high in some states, with Kebbi State having the highest prevalence of 52.2% in 2018 ^2^, despite the nation’s concerted efforts aimed at eliminating malaria. Various malaria control strategies deployed by the National Malaria Elimination Programme (NMEP) in Nigeria have failed to reach some of its previous goals and are unlikely to achieve its current goal of parasite prevalence of less than 10% in the country ^3^. Several factors may drive the inability to achieve these goals.

Sociodemographic factors significantly influence malaria prevalence, transmission, and the effectiveness of control measures within communities. Age is one of the most critical determinants, with young children and pregnant women being particularly vulnerable. Their weaker immune systems make them less able to fight the malaria parasite, leading to higher morbidity and mortality rates in these groups. There is a continual need for public health initiatives geared towards prioritising this vulnerable population by providing interventions such as insecticide-treated nets (ITNs), intermittent preventive treatment in pregnancy (IPTp) and routine health education campaigns tailored to their needs ^4– 6^. Socioeconomic position directly or indirectly determines malaria exposure and outcome. People from lower-income households often live in poorly constructed homes that lack basic mosquito-proofing features such as window screens or sealed walls. They may not be able to afford certain preventive tools, diagnostic tools, or adequate, timely medical care when infected. There could also be social exclusion or lingering malnutrition, which can contribute to susceptibility to malaria infection and disease progression ^7– 10^. These conditions perpetuate a cycle in which poverty and high malaria prevalence reinforce one another, underscoring the need for affordable healthcare and targeted subsidies in malaria control programs ^11^.

Education also plays a pivotal role in shaping attitudes and behaviours toward malaria prevention. Individuals with higher education levels are more likely to understand the importance of protective measures, and as such, community education programs are essential in bridging the gap caused by the difference in educational level, thus promoting healthier behaviours ^9, 11, 12^. Cultural norms and gender roles also influence malaria dynamics in many communities. For example, women may face barriers in making healthcare decisions or accessing preventive tools, especially in patriarchal societies. Recognising and addressing these sociodemographic determinants is vital for designing targeted and effective malaria interventions. Tailored strategies that account for these factors can significantly improve outcomes and reduce the burden of malaria in vulnerable populations ^13– 15^.

This study aimed to investigate the prevalence of asymptomatic malaria at the end of the rainy season and identify sociodemographic determinants of malaria infection in communities in Nnewi North LGA, which is in Anambra State. We have good surveillance data for malaria in children under 5 years old compared to other age groups in Nigeria. Anambra has the second-lowest prevalence of malaria in children under 5 years of age, compared to other states in Nigeria. Malaria prevalence has continued to decrease in Anambra State from 2010 to 2018, with a 5.4% decrease in prevalence in 2018 vs 2010 ^2^. It is not certain if this decline has been sustained, as there was no data on malaria prevalence in the 2023–24 Nigeria Demographic and Health Survey; rather, a proxy measure of diagnostic testing for malaria was assessed in children who had fever 2 weeks before the survey ^16^. A recent ongoing study ^17^ has indicated that severe malaria outbreaks occur during the rainy transmission season, which is between April and October. Therefore, there may be a higher burden of residual parasite load among the asymptomatic adult population that could contribute to the sustained transmission of malaria parasites. If this burden is higher, it may be reflected at the end of the rainy season and continue to the next transmission season.

We intended to uncover and analyse the socioeconomic factors and health-seeking attitudes and beliefs influencing the prevalence of asymptomatic malaria that continues to sustain the transmission of the malaria parasite in Anambra State. Systematically investigating these potential factors through an observational approach can provide evidence that could support the development of evidence-based targeted malaria control programs in Anambra State and other parts of Nigeria.

## Methodology

### Study design

This was a cross-sectional study that utilised a questionnaire survey to collect qualitative, independent, and explanatory variables and malaria rapid diagnostic test (mRDT) kits to determine the outcome variable, which was asymptomatic malaria in adults living in Nnewi North LGA. Regression analysis was used to determine which of the qualitative variables is protective of asymptomatic malaria in Nnewi North LGA, Anambra State. 

### Study area

**Figure 1.**
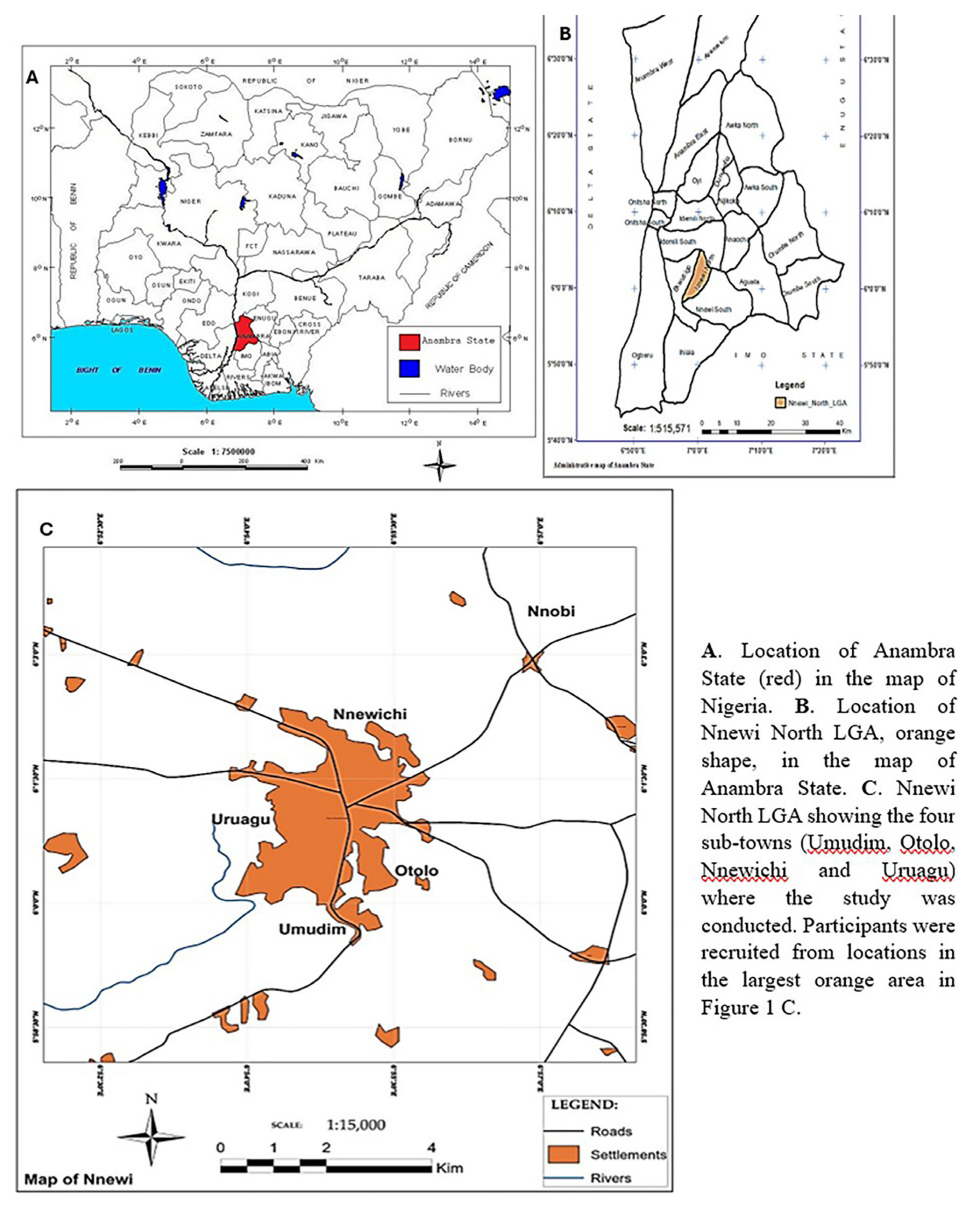
Map of Nnewi North Local Government Area in Anambra State, original images were provided by the author and were slightly modified for clarity ^21^. This figure/table has been reproduced with permission from [Chukwukalo Ezeomedo I, Izuchukwu Igbokwe J. Mapping of Urban Features of Nnewi Metropolis Using High Resolution Satellite Image and Support Vector Machine Classifier. 2019].

This study was conducted across the four sub-towns ( Figure 1A) of the Nnewi North LGA, Figure 1B of Anambra State, and the southeast geopolitical enclave of Nigeria. The Nnewi kingdom was founded in four quarters (large villages or sub-towns): Otolo, Uruagu, Umudim, and Nnewi-Ichi. Most people in this area are of the Igbo ethnic origin. The commonly spoken language is Igbo, followed by English. Most people here practice Christianity. Nnewi is home to many major indigenous manufacturing industries, including the Ibeto Group of Companies, Cutix and AD Switch, Uru Industries Ltd, and Omata Holdings Ltd. Most industrialists in the cluster of spare parts factories in Nnewi are also traders. Major trading centres include the Nkwo Nnewi, Nwafor, and Eke Ichi markets. The traditional ruler of Nnewi North LGA is referred to as the Igwe of Nnewi, and popular community festivals include the Afiaolu and Ofala festivals. Nnewi hosts several institutions and places for learning and healing, including Nnamdi Azikiwe University Teaching Hospital (NAUTH). NAUTH provides specialised and comprehensive medical care ^18, 19^. Domestic water supply is a big challenge with water available from borehole pumps, overhead/ground tanks and other water storage containers. Malaria is endemic and perennial in Anambra, with transmission occurring mainly from April to December for approximately 9 months ^20^.

### Participants

Participants were recruited using a stratified purposive sampling technique from markets, banks, offices, and churches within sub-towns. The sub-towns served as strata. This technique ensured diversity in terms of socioeconomic status and access to healthcare. Recruitment was performed after obtaining ethical approval, permission from the chairman of the Nnewi North LGA, and consent from each participant. Recruitment was conducted for two weeks in November, from 7 November 2024 to 20 November 2024, at the end of the rainy season.

Participants were considered eligible for inclusion if they were healthy, 18 years of age or older, and living in the sub-towns of Nnewi North LGA in Anambra State for at least the past 3 years. People who had lived outside a malaria-endemic country for most of their lives, and within the last three years, and those who did not give consent were excluded. A balanced representation of participants from urban and rural areas was ensured.

### Data collection/measurements

A detailed, structured, and validated questionnaire Consent form and RESEARCH QUESTIONNAIRE_Malaria_Epi Study), comprising 19 items divided into six sections assessing the participant's demographics (seven items), knowledge of malaria disease (five items), knowledge about malaria prevention (13 items), knowledge of malaria treatment (two items), perception, attitudes, and beliefs towards malaria (17 items), and community activity towards malaria (five items) was developed to collect information on the independent variables from the participants during the study. The questionnaire was presented in English and was self-administered or researcher-administered if the respondent could not fully understand English. Pre-tests and re-tests were conducted with four volunteers who were not part of the study to determine the reliability of the questionnaire items before the questionnaire was finalised. The pre-test was carried out on the 12th of September 2024, and the retest was carried out on the 18th of September 2024. Face validity was assessed by an expert and a non-expert in epidemiology. The overall Cronbach's alpha for the questionnaire during the pretest was 0.88 ( Pre-Test and Re-Test), indicating good internal consistency. Cohen's kappa values were calculated to assess the reliability of the responses between the pre-test and retest for each section of the questionnaire. Overall, the questionnaire demonstrates good to excellent reliability, with Kappa values consistently falling between 0.72 and 0.82, indicating strong consistency in responses across the pre-test and re-test. The questionnaires were modified, as suggested, before they were included in the application for ethical approval. Consent was obtained before obtaining any data or blood samples.

A finger prick was used to obtain 5 μl of blood from the respondents to detect the presence of infection with *P*. *falciparum* using a rapid lateral flow immuno-chromatographic *in vitro* antigen detection test kit for detecting *P. falciparum* HRP2 (histidine-rich protein 2) (First Response Malaria Antigen *P. falciparum* (HRP2) Card Test (100% Sensitivity and 100% Specificity) by Premier Medical Corporation Limited and Standard Q Malaria P.f Ag (99.38% Sensitivity and 100% Specificity) by SD BIOSENSOR) according to the manufacturer’s instructions. The results were read within 20 minutes, during which time the respondents were able to complete the questionnaire.

### Bias

This study was prone to selection bias, as the sample of this population who could not give 20 minutes of their time were excluded from the study. This study was also prone to recall bias, as some participants may have recalled past events differently. The researcher-administered questionnaire may have prompted favourable answers, thus modifying our primary data in some way.

### Study size

This study aimed to determine the prevalence of malaria among adults in Nnewi North LGA and the associated social, economic, environmental, and behavioural determinants. The sample size was calculated based on the prevalence of malaria in children under 5 years of age. This was appropriate because we intended to determine whether the prevalence in a population that serves as a reservoir for continual transmission is related to the prevalence in individuals under 5 years of age. The latest Nigeria Demographic and Health Survey (NDHS) conducted in 2018 showed that the prevalence of malaria in children under 5 years in Anambra State was 8.8% ^2^. The prevalence data obtained for children during the national survey in Anambra were used to calculate the sample size of the adults in this study.

The sample size was determined using the simple Cochran sample size formula ^22^: 

*n* = *Z* ^2^ *P*(1− *P*)/ *d* ^2^

where n = sample size, Z = Z statistic for a level of confidence (1.96), P = expected prevalence or proportion (0.088*), and d = precision (0.05; 95% CI). n was calculated to be 123, and when we allowed for 10% attrition, the sample size was 137 (123/0.9). In total, 140 questionnaires were used in this study. 35 questionnaires were to be used per sub-town, but we used slightly more in some sub-towns due to non-responses and time constraints.

### Ethical approval

Ethical approval for the study was obtained from the Ethics Committee, Nnamdi Azikiwe University Teaching Hospital (NAUTH) – NAUTH/CS/66/VOL.17/VER.3/103/2024/081- on 9th October 2024, ( ethical approval). Written informed consent was sought from all participants, and the data were de-identified using the ‘Safe Harbour’ method and are publicly available ( Questionnaire responses (ALL ITEMS)_ver 2. The participants were informed of their right to withdraw from the study at any time.

### Data analysis

The obtained data were analysed using Stata 17/BE, and significant associations were measured at the 5% alpha level (p < 0.05). Descriptive statistics were used to summarise the data, while inferential statistics (chi-squared tests and logistic regression) were used to identify significant associations between the factors studied and malaria prevalence. Qualitative data were analysed to identify recurring patterns and insights that complemented the quantitative findings. Bivariate statistical analyses were performed. Bivariate analysis - Chi-square (χ2) - was used to determine significant dependence between the categorical independent variables and malaria-positive outcomes. The independent variables with a significant relationship with malaria were fitted into the binary logistic regression model, as the dependent variable, malaria status, is binary. The results of logistic regression are presented as odds ratios (OR). These ratios represent the magnitude of the malaria risk. OR, also known as crude odds, represents the likelihood of an event when other variables are not taken into consideration. The likelihood of contracting malaria increases when the OR is higher than one (OR > 1). In situations where the OR is less than 1 (OR < 1), the chance of malaria is reduced. All results were presented as 95% confidence intervals. 

### Variables

***Outcome variable.*** The dependent or outcome variable in this study was the asymptomatic malaria status determined through malaria rapid diagnostic tests (mRDT). This variable was defined in a binary manner, with a positive malaria result of 1 and a negative result of 0.

***Independent potential explanatory variables.*** These include sociodemographic characteristics, socioeconomic factors, healthcare-seeking behaviour, use of malaria prevention measures, environmental conditions, and perceptions of malaria risk, obtained through answers to the items in the questionnaire. These independent variables have already been categorised. All responses were double-checked, and some were converted into dummy variables for modelling purposes.

## Results

### Study participants

A total of 160 adults were informed of the aim of the study and approached, and 140 adults were eligible for the study. Of the 140 patients who were confirmed as eligible and gave their consent to be included in this study, only 130 completed the questionnaires and were tested for malaria parasites using the mRDT, giving a response rate of 92.86%. However, with this response rate, we met our enrolment target of 127 participants. Data from all subjects were analysed. At every stage of our recruitment process, non-response was due to exclusion criteria, time constraints and phobias of needles on the part of the potential participants, even after they were calmed.

### Demographic and socioeconomic characteristics of the study participants

Most participants were aged between eighteen and thirty-seven years, Table 1, followed by those who were thirty-eight to fifty-seven years. Very few (7) participants above seventy-eight years old were recruited into the study. More males than females participated in the study. Very few (4) persons with no formal education were recruited, and trading was the most popular profession among this adult population, followed by farming, and then being a student. In addition, a few participants were working in a professional role and as civil servants, even though more participants had attained post-primary education, with those attaining secondary education being the highest. The participants resided mainly in a rural area, with most living in households of one to four persons, including themselves, followed by those living in households of five to eight persons. We tried to equalise the number of participants from each sub-town, but more people from Nnewichi and Umudin participated in the study. However, the number of participants was evenly distributed across the sub-towns.

**Table 1.** Demographic and socioeconomic features of the participants.

Variable	No. recruited	Malaria prevalence * (%)	X ^2^ (P)
**Age**			**3.48 (0.32)**
18 – 37	68	26.47	
38 – 57	35	17.14	
58 – 77	20	40.00	
Above 78	7	28.57	
**Gender**			**0.46 (0.50)**
Male	70	28.57	
Female	60	23.33	
**Highest Level of Education**			**2.09 (0.55)**
Non-Formal Education	4	0.00	
Primary Education	25	28.00	
Secondary Education	61	29.51	
Tertiary Education	40	22.5	
**Occupation**			**7.08 (0.31)**
Pupil/Students	18	33.33	
Trader	43	32.56	
Farmer	31	22.58	
Civil-Servant	14	0.00	
Professional	5	40.00	
Menial Labour	12	25.00	
Unemployed	7	28.57	
**Type of Residence**			**0.02 (0.90)**
Urban Area	60	26.67	
Rural Area	70	25.71	
**NUMBER OF HOUSEHOLD RESIDENTS**			**0.62 (0.74)**
1 – 4 Persons	75	25.33	
5 – 8 Persons	53	26.42	
9 – 12 Persons	2	50.00	
13 persons and Above	0	0.00	
**SUB-TOWN**			**1.67 (0.65)**
Nnewichi	36	19.44	
Otolo	28	32.14	
Umudim	37	29.73	
Uruagu	29	24.14	

* Malaria prevalence was normalised to the number of participants in the subgroups of various categories. This means that within a subgroup, the malaria prevalence recorded is for that subgroup and not with reference to the total number of participants.

### Prevalence of asymptomatic malaria amongst study participants

**Figure 2.**
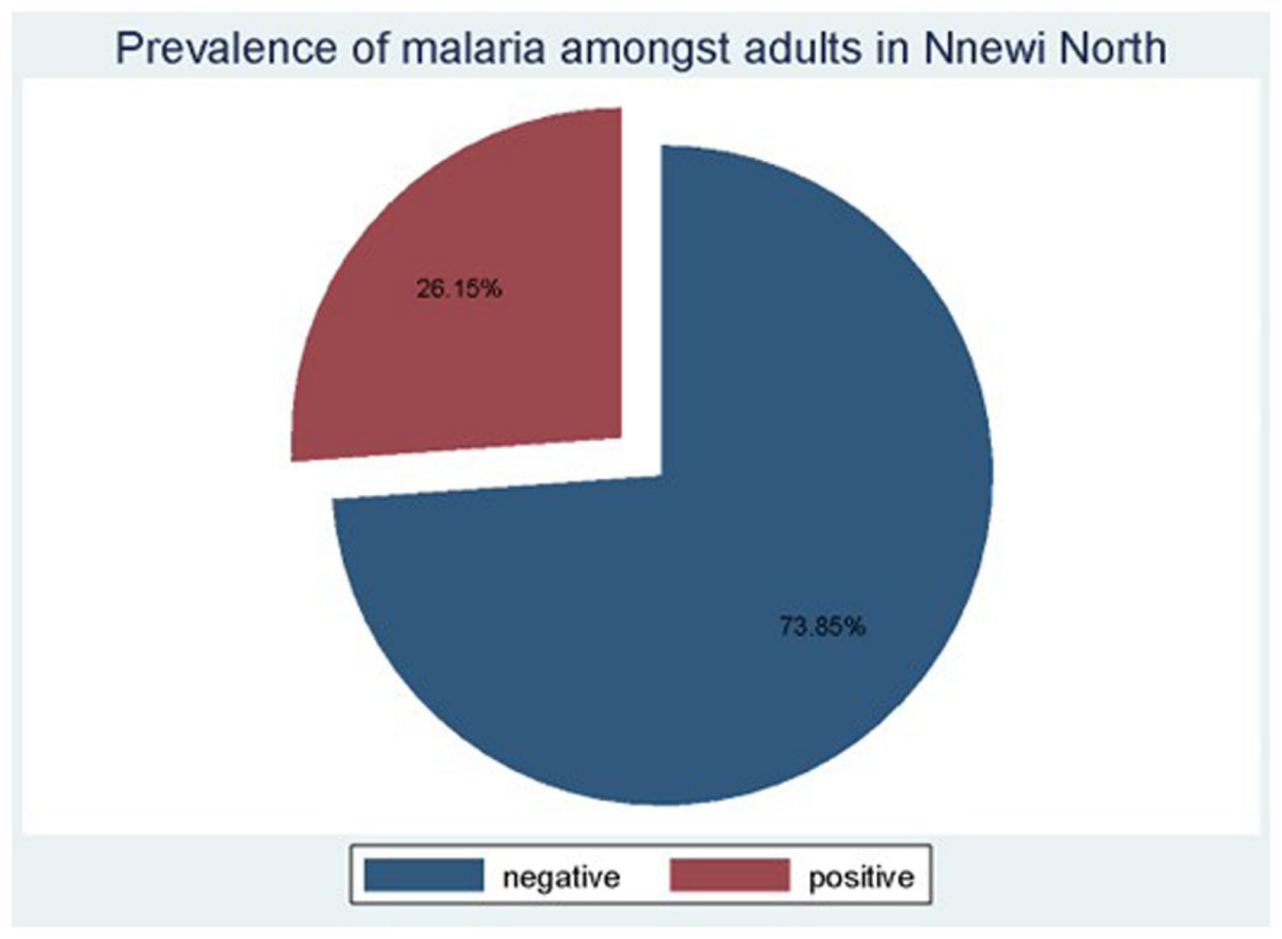
Prevalence of malaria amongst asymptomatic adults in Nnewi North LGA of Anambra State.

Of the 130 participants examined for malaria infection using the mRDT, 34 tested positive, thus giving a prevalence rate of 26.15%, as shown in Figure 2. All parasites detected were *P*. *falciparum*, using an HRP2-based mRDT kit.

The major contributors to the prevalence of asymptomatic malaria among the different demographics and socioeconomic categories were adults of fifty-five to seventy-seven years of age, Table 1, adults with professional jobs, adults living in urban areas, adults living in households with nine to twelve occupants, and residents from Otolo sub-town, even though the number of participants in these sub-groups was smaller in the category. Other subgroups majorly contributing to the malaria prevalence in this community include males and those with secondary education, even though their numbers were smaller compared to other subgroups of their category.

### Participants history of and knowledge about malaria

Most of the participants (60.77%) had malaria in the year of study recruitment ( Table 2). Participants who had previously had malaria in the year contributed more to the prevalence of asymptomatic malaria than those who did not have malaria in the same year, but the difference between these subgroups was not significant. All participants were aware of malaria as a disease.

**Table 2.** Participants knowledge and history of malaria disease.

Variable	No. recruited (%)	Malaria prevalence * (%)	X ^2^ (P)
**Are you aware of malaria?**			
Yes	100	26.15	
No	0	0.00	
**Have you suffered from malaria since this** **Year?**			**0.9136 (0.34)**
Yes	79	29.11	
No	51	21.57	

* Malaria prevalence was normalised to the number of participants in the subgroups of various categories.

**Figure 3.**
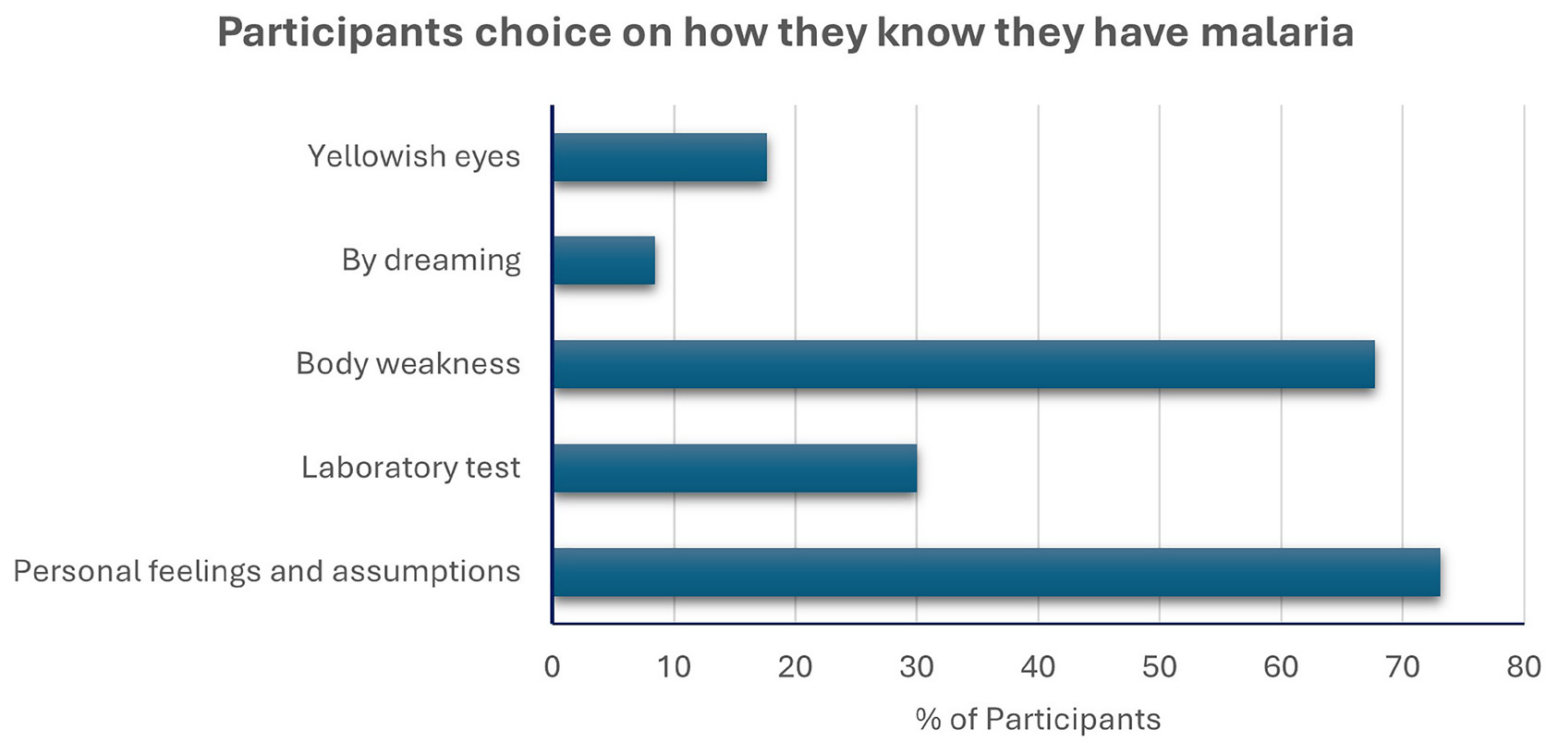
Participants’ knowledge of how they know they have malaria. Participants were allowed to select multiple options. The cumulative percentage is more than 100%.

**Figure 4.**
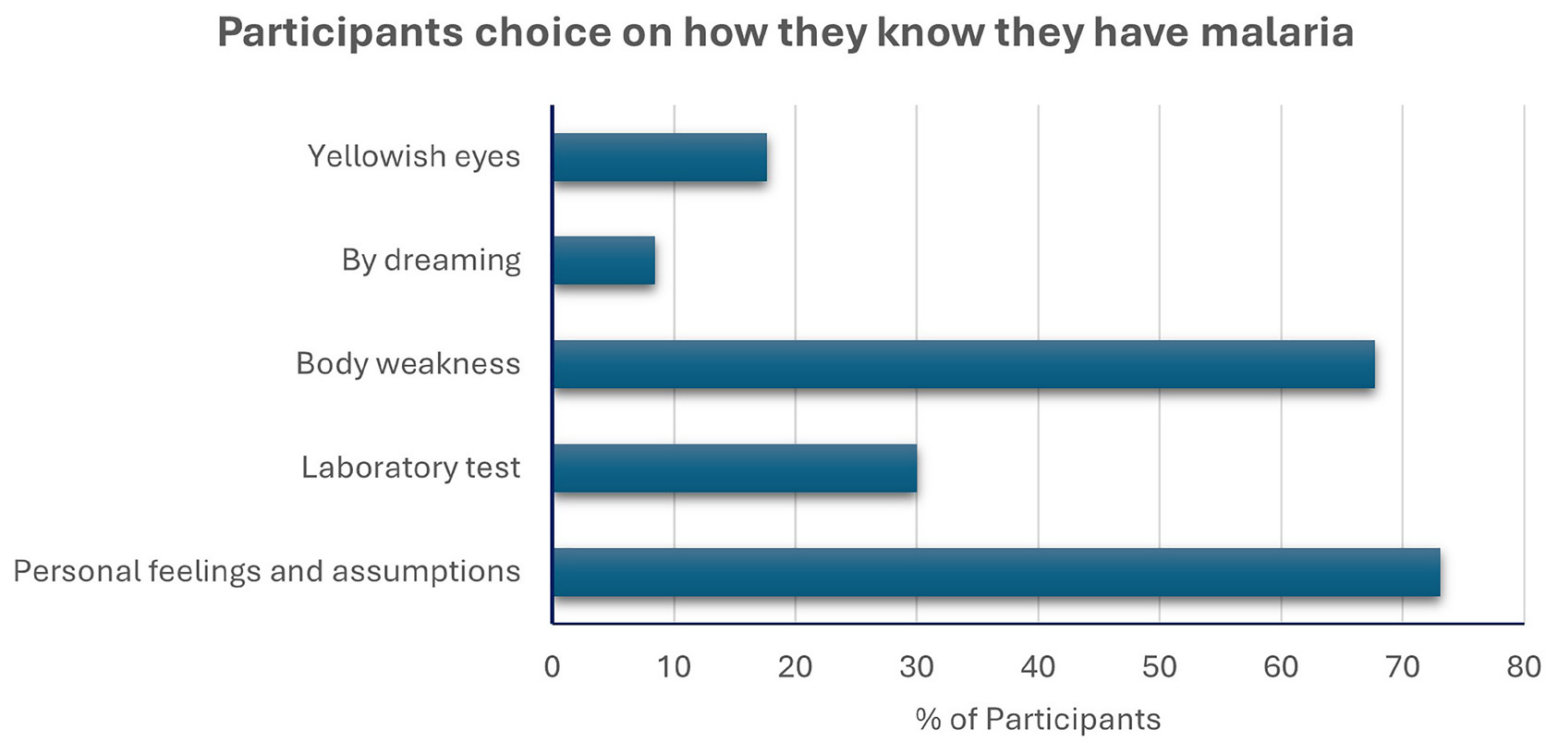
Participants’ knowledge of malaria transmission and malaria symptoms. A: Knowledge of how malaria is transmitted. B Knowledge of malaria symptoms. All the presented modes of transmission and symptoms of malaria were already contained on the questionnaire, and the participants were allowed to select multiple options.

However, the data on previous malaria may be influenced by their knowledge of the signs and symptoms, and transmission of malaria. Figure 3 shows that most participants relied on personal feelings and assumptions as the criteria for determining that they had malaria, with a minority relying on confirmatory laboratory tests. In contrast, most participants know how malaria is transmitted, with the majority selecting mosquitoes as the mode of transmission ( Figure 4A). Similarly, most of them selected the common symptoms of malaria (Figure 4B)– fever, body weakness, headache, shivering and body pains or muscle aches.

For preventive strategies, we have a greater contribution to malaria prevalence from those who never or rarely use insecticide-treated bed nets ( Supplementary data: Supplement 1: Participants' utilisation of malaria prevention methods. The greatest contribution is from those who often use mosquito repellents/coils. Those that rarely sprayed insecticides contributed the most to the prevalence of malaria, whereas those that never fumigated their environment were the greatest contributors. Counterintuitively, for some preventive activities, increased frequency of that activity did not translate into a reduced prevalence of asymptomatic malaria. This was reflected in the use of antimalarial chemoprevention, nets on the windows, or prayer. The findings presented for these preventive strategies thus far have not been found to be significant.

There was a significant relationship between the malaria preventive strategy ‘Covering Water Containers’ and asymptomatic falciparum malaria, see Supplementary data: Supplement 1: Participants' utilisation of malaria prevention methods. Those who always covered water containers did not have asymptomatic falciparum malaria. This created a problem of “separation” when fitting a binary logistic regression model for this category. However, when the frequency of covering water containers was further analysed using the Firth’s penalised maximum likelihood regression model, the odds of having asymptomatic malaria were not significantly reduced when water containers were always covered, Table 3. The apparent dependence of asymptomatic malaria on covering water containers, previously observed using the Chi-square test, may be due to the confounding effect of other covariates.

**Table 3.** Covering water containers as a malaria preventive strategy.

Variable	Odds Ratio	95% CI
**Covering water containers**		
Never	Ref	
Rarely	0.380	0.078 – 1.844
Sometimes	1.485	0.423 – 5.216
Often	1.000	0.267 – 3.743
Always	0.081	0.004 – 1.662

The odds of having asymptomatic malaria when different frequencies of covering water containers are applied. p value for the ‘Always’ group was 0.103.

During a malaria episode, most participants would use antimalarial drugs Supplementary data: Supplement 2: Knowledge and attitude for malaria treatment, and did contribute less to asymptomatic malaria; however, their contribution to asymptomatic malaria was not significantly different compared to those that did not use antimalarial drugs. Herbal remedies were commonly used, and those who used them contributed less to asymptomatic malaria. Using local concoctions, taking lots of fruit, prayers, and visiting the hospital made no difference to the carriage of *P. falciparum*. None of the three participants who did nothing had asymptomatic malaria at the time of testing. None of the differences were statistically significant.

There is no consensus about the effectiveness of most malaria control strategies deployed in the communities in Nnewi North LGA ( Supplementary data: Supplement 3: Perception, attitudes and beliefs towards malaria control). This lack of consensus may not correspond with asymptomatic malaria. For example, most participants agreed or strongly agreed that sleeping under an insecticide-treated net (ITN) effectively prevented malaria, but those who strongly agreed contributed the most to asymptomatic malaria, showing a discrepancy in belief and practice. On the other hand, those who strongly disagreed that their community was actively involved in malaria prevention efforts contributed the most to asymptomatic malaria. Only one range of perceptions and beliefs was different and concurrent. This is shown in the agreement that ‘the cost of malaria prevention tools, such as Insecticide-treated nets (ITNs), insecticides and mosquito repellents, is reasonable’. Those who believed this contributed significantly less to asymptomatic malaria; with those who agreed or strongly agreed having a lower odds of having asymptomatic malaria, with an odds ratio of 0.29 or 0.27 (95% CI = 0.07/0.06 – 1.24), compared to those who strongly disagreed, Table 4.

**Table 4.** Individual and community malaria control strategies.

Variable	Odds Ratio	95% CI
**The cost of malaria prevention tools, such as ** **Insecticide-treated nets (ITNs), insecticides ** **and mosquito repellents, is reasonable.**		
Strongly disagree	Ref	
Disagree	1.47	0.37 – 5.86
Neutral	0.60	0.16 – 2.33
Agree	0.29 *	0.07 – 1.24
Strongly agree	0.27 *	0.06 – 1.24
**Do you think the malaria level is low in your ** **Community?**		
Yes	Ref	
No	2.51 **	1.03 – 6.12
Don’t know	0.78	0.19 – 3.26

For statistical significance, *p < 0.1 to ≥ 0.05. **p < 0.05

Importantly, most participants were optimistic that malaria could be eradicated from their community, but did not think that the malaria level in their community was currently low ( Supplementary data: Supplement 4: Community activity towards malaria control, and they had a higher odds of having asymptomatic malaria ( Table 4). It was encouraging that most of them knew that their community faces challenges in preventing and treating malaria and that they need to do more to prevent the transmission of malaria.

## Discussion

Malaria transmission in Anambra State is perennial, with a much higher prevalence during the rainy season than during the dry season ^23^. Asymptomatic *Plasmodium* infection contributes to the continuous transmission of malaria parasites. In this study, we determined the prevalence of asymptomatic malaria to be 26.15% in adults living in the Nnewi North Local Government Area at the end of the rainy season. This prevalence is about triple the prevalence of malaria in individuals under 5 years of age in Anambra State, as determined during the latest NDHS ^2^. Asymptomatic infection occurs after childhood when varying levels of partial immunity to malaria have developed, such that the infection still thrives, but progress to a disease state is minimised. The prevalence of asymptomatic malaria among the adult population here is similar to that in Ido-Ekiti, southwest Nigeria ^24^, but is much higher than the prevalence in another community, Aguleri, in Anambra State, where the prevalence was determined to be 5.3% in the adult population of 20 years and above ^25^. Strategies geared towards malaria control must consider factors related to the host, the malaria parasite, the malaria parasite–transmitting mosquito vector, the environment, and the health systems capacity in such environments ^20^. The agreement that the cost of malaria preventive tools was reasonable was associated with a reduction in asymptomatic malaria. This study focuses on other factors related to the host, environment, and some community-level aspects of health system capacity.

Our study participants consisted mostly of adults aged between eighteen and thirty-seven years, and there were more males than females. Trading was the most popular profession in the Nnewi North LGA, and most people had some form of formal education, mainly at the secondary school level. These factors had no bearing on the prevalence rate of asymptomatic malaria detected in the community, nor did residing in rural areas or crowded households impact this rate. None of these demographic or socioeconomic factors explained the prevalence of asymptomatic malaria. Most of the participants claimed to have had malaria in the year of the study recruitment, but most of them relied on personal feelings and assumptions as signs of malaria. This points to a critical gap in the strategies deployed for malaria elimination, as the signs and symptoms of malaria are similar to those of other diseases, even though most of them could correctly identify its signs and symptoms. Effective and proactive community health education is needed to steer the populace, including healthcare professionals, towards supporting the correct and complete diagnosis of malaria before treatment is administered.

A measure of community health education by assessing the participants’ knowledge of malaria control revealed that all the participants were aware of malaria as a disease. They were also aware of and utilised various preventative measures. In a systematic review of data from studies of different preventive control measures, ITNs, indoor residual spraying (IRS), prophylactic drugs (PD), and untreated nets (UN), only ITNs were shown to be highly effective ^26^. In a study in Nigeria, the lack of formal education, diabetes, and ITNs were the specific determinants of asymptomatic malaria ^24^. However, in the present study, even with the increased utilisation of various preventive measures, covering water containers could be an effective preventive measure for asymptomatic malaria. Covering water containers reduces the availability of habitats required for the maturation of mosquito larvae. This is termed larval source management (LSM) ^27^, and in this case, it physically disrupts mosquito breeding. LSM has been shown to reduce parasite prevalence in several countries, including Sri Lanka, India, the Philippines, Greece, Kenya, and Tanzania ^27^. Additionally, drinking water and sanitation are important risk factors for malaria infection in children under 5 residing in sub-Saharan Africa, irrespective of their socioeconomic status ^28^. Indeed, a qualitative study utilising focus group discussions with key stakeholders has pointed to environmental sanitation, including LSM, as a ‘game changer’ for malaria elimination, but it will require effective collaboration and political will that also involves organised communal labour activities ^29^.

In Nigeria, the major mosquito vectors responsible for malaria are two species of the *Anopheles gambiae* species complex or *Anopheles gambiae* sensu lato (s.l.) – *An. gambiae* sensu stricto (s.s.) and *An. Arabiensis*, and a member of the *An. funestus* species complex - *An. funestus* s.s.. *An. gambiae* (s.s.) is the predominant vector in Anambra State ^30, 31^. There is a higher distribution of *An. gambiae* s.s. than *An. arabiensis* in the wet season ^31^. *An. gambiae* and *An. funestus* thrive in rural areas and are rarely found in highly urbanised regions. Another mosquito vector, *An. stephensi,* which thrives in cities and towns, is becoming an important vector for malaria transmission in Nigeria ^32– 34^ due to an increasing urbanisation. This specific vector is known to breed in man-made containers, including plastic drums and water tanks ^35^, which are commonly used in Nnewi North LGA of Nigeria. In this study, the higher prevalence of asymptomatic malaria compared to the prevalence of malaria in children who are under 5 years old may point to possible spread of *An. stephensi* in Nnewi North LGA and other surrounding urban regions in Anambra State.

The use of antimalarial chemotherapy is widespread in Nnewi North LGA; however, herbal remedies are also being used. In fact, those who used herbal remedies for malaria contributed less to asymptomatic malaria. *In vivo* animal studies comparing crude herbal extracts with artesunate demonstrated that herbal extracts can enhance ^36^ or inhibit ^37^ the antimalarial activity of artesunate. This means that the effectiveness of ACTs can be affected by herbal remedies, and this effect could lead to ACT drug failure. The effect of such a combination is entirely dependent on the constituents of the herbal remedies, which is hardly ever clear. Therefore, it is safer not to combine them.

Public health education and intervention should involve reinforcement of any strategy that should be implemented at the individual level, as there seemed to be a difference between what participants believed and what they practised. This is exemplified in the belief that the cost of Insecticide-treated nets (ITNs), insecticides, and mosquito repellents was reasonable, likely contributing to their increased purchase, usage and protection, as there was a reduced odds of having asymptomatic malaria. Empowering people to make healthier choices will reinforce individual and community-based malaria control efforts. The participants did not think that the malaria level was low in their communities, but were generally hopeful that malaria could be eradicated from their community, despite the challenges in implementing preventive measures and malaria chemotherapy. Participants were also willing to do more to eliminate malaria. This willingness can be fashioned into an effective control strategy when combined with economic empowerment and political will.

This study is limited by recall bias and the recruitment strategy, which will likely exclude those who were not accessible, adults who do not frequently visit public places, and those who are unemployed. Only 7 participants were unemployed. The researcher-administered questionnaire may have prompted favourable answers, thus modifying our primary data in some way. Future studies will utilise questionnaires that are translated into the most common language spoken in the study area. To enhance inclusivity and external validity, random sampling techniques and home-based recruitment can be used to include a more diverse participant pool. As the uptake of malaria control commodities and implementation of malaria control strategies increases in various communities, it would be important to see their effect on the number of asymptomatic malaria cases in the future.

## Conclusion

The prevalence of asymptomatic malaria among semi-immune adult respondents living in Nnewi North LGA is three times higher than that of children under 5 years in Anambra State, according to the most recent NDHS. Ongoing community health education is essential to ensure proper diagnosis and treatment of malaria. ‘Prevention is better than cure’, as the saying goes, and covering water containers around the home is a suitable preventive strategy for asymptomatic malaria in Nnewi North LGA. LSM, which includes covering water containers. This can be promoted alongside other control measures. Future research may focus on identifying the most effective, cost-efficient, and easily implementable LSM practices. Economic empowerment can help to reinforce the utilisation of malaria preventive strategies, as the affordability of malaria prevention goods can translate into a reduction in asymptomatic malaria. A combination of strong political commitment, economic empowerment and community effort will be key to successfully fighting malaria. Nnewi North LGA is committed to participating in the fight against malaria. We have shared our findings with the Anambra State National Malaria Elimination Programme, which could be useful for making programmatic decisions.

## Ethics and consent

The study protocol was reviewed and approved by the NAUTH Ethics Committee. Full ethical approval, with number - NAUTH/CS/66/VOL.17/VER.3/103/2024/081- on 9th October 2024, before the study commenced. Written informed consent was obtained from the respondents for the study, and for the storage and publication of the collected data, before they participated in this study. All institutional and research ethics guidelines, rules, and regulations were followed in this study.

## Appreciation

We would like to thank the staff and students of the Department of Pharmaceutical Microbiology and Biotechnology, Nnamdi Azikiwe University, for their academic or other support.

## Data Availability

Figshare: Covering water containers is a strong preventive measure for the reduction of asymptomatic malaria towards the end of the rainy season in Nnewi North Local Government Area of Anambra State, Nigeria, https://doi.org/10.6084/m9.figshare.28881803.v2 ^38^. This project contains the following underlying data: PRE-TEST AND RE-TEST _ malaria _ epi study. (Reliability test of the questionnaire items). Consent forms and RESEARCH QUESTIONNAIRE_Malaria_Epi Study.pdf (form used to obtain consent and the unfilled questionnaire). ETHICAL APPROVAL Malaria_Epi Study.pdf Questionnaire responses (ALL ITEMS))_ver 2.xlsx (compiled raw data) Figshare: Covering water containers is a strong preventive measure for the reduction of asymptomatic malaria towards the end of the rainy season in Nnewi North Local Government Area of Anambra State, Nigeria, https://doi.org/10.6084/m9.figshare.28881803.v2 ^38^. Supplementary data.docx (Extended data) The data are available under the terms of the Creative Commons Attribution 4.0 International license (CC-BY 4.0).
