# Supplementary material for: Economic empowerment and various preventive strategies play a role in reducing asymptomatic malaria towards the end of the rainy season
Source: Open Res Afr. 2025 Oct 6;8:5. Originally published 2025 Jun 18. [Version 2] doi: 10.12688/openresafrica.15809.2 (PMC12375191; doi:10.12688/openresafrica.15809.2)
Supplement: Peer review history

# Reviewer report: Reviewer response for version 2

**DOI:** 10.21956/openresafrica.17408.r32645

**Published:** 2025-10-11

**Authors:** Johanna P Daily

**Affiliations:** 1 Albert Einstein College of Medicine, New York, USA

No further comments on this manuscript

Is the work clearly and accurately presented and does it cite the current literature?

Yes

If applicable, is the statistical analysis and its interpretation appropriate?

Partly

Are all the source data underlying the results available to ensure full reproducibility?

Yes

Is the study design appropriate and is the work technically sound?

Yes

Are the conclusions drawn adequately supported by the results?

Partly

Are sufficient details of methods and analysis provided to allow replication by others?

Yes

Reviewer Expertise:

malaria

I confirm that I have read this submission and believe that I have an appropriate level of expertise to confirm that it is of an acceptable scientific standard.

# Reviewer report: Reviewer response for version 1

**DOI:** 10.21956/openresafrica.16984.r32300

**Published:** 2025-08-25

**Authors:** Allan Schapira

**Affiliations:** 1 Bicol University College of Medicine, Legazpi City, Philippines

The conclusion, already in the title, suggests that a causality has been demonstrated by the study.  However, it is  cross-sectional study; it can therefore only identify associations. 

 The interesting association between thinking that covering containers helps control malaria and low parasite prevalence is supported by data found only in a Supplement. This is unacceptable: All the data from the Supplement (1 and 2) should be included in the article.

 It is not universally accepted that asymptomatic parasitemia (in adults) is the main driver of transmission. This contention should be supported by relevant literature and more information on malaria transmission in the State. If there is strong seasonality with interrupted transmission for several months, the contention is more plausible.

 Nothing is known about the practices of the study population. All the independent variable data is about what they say they think. We don’t know whether they do what they think is useful. The association between saying that covering water containers is useful and low prevalence might be confounded by better education, better income, better housing etc.

 With a total of 21 independent variables examined in Supplement 1 and 2, the finding of a single statistically significant association (p=0.03) cannot be accepted as evidence that there is an association, only that there may be an association. Pure chance could easily provide a p<0.05 for 1 or 2 variables. 

 However, there might be an association. And it MIGHT be that this association is not just due to confounding as suggested above. The issue merits further analysis which unfortunately is missing. The main malaria vectors in Nigeria are *An. gambiae, An. arabiensis and An. funestus.* These rarely breed in containers (Af probably never).  However, in recent years, *An. stephensi *, which mainly breeds in containers has emerged in some places in Nigeria. A priori it is unlikely to have come to play a main role in the study area, because if it had, malaria transmission would be expected to increase, not decrease over the last 10 years.

 The authors should present their results to the State health authorities and ask them how their finding matches or not with the entomological situation. They may think that is outside their remit as academic researchers. The opposite is the case. Scientific work examines the context and looks for explanations for unexpected findings. Mere presentation of data with p-values is not science.

Is the work clearly and accurately presented and does it cite the current literature?

No

If applicable, is the statistical analysis and its interpretation appropriate?

Partly

Are all the source data underlying the results available to ensure full reproducibility?

Yes

Is the study design appropriate and is the work technically sound?

Yes

Are the conclusions drawn adequately supported by the results?

No

Are sufficient details of methods and analysis provided to allow replication by others?

Yes

Reviewer Expertise:

NA

I confirm that I have read this submission and believe that I have an appropriate level of expertise to state that I do not consider it to be of an acceptable scientific standard, for reasons outlined above.

# Response

**Published:** 2025-10-07

**Authors:** Linda Onyeka Anagu

Dear Allan Schapira,

 The conclusion and title have been modified.  

 Your suggestion that all the data from the Supplement (1 and 2) should be included in the article is valid, but in this case, I have only presented the data on covering water containers as a figure in the article, as the rest of the data adds very little information.  

 Supplement 1 has been correctly titled participants’ utilisation of malaria prevention methods, as our questions targeted their use of the assessed methods. Potential confounders like better education, better income, better housing, etc, of the observed association have been noted. Further analysis has been done to confirm the association between covering water containers and asymptomatic malaria using the Firth's method to overcome the problem of "separation" in logistic regression, as ‘Always covering water containers’ perfectly predicts not having asymptomatic malaria, as shown in Supplement 1. The results of this analysis have been reported in the modified manuscript. We have written up a policy brief and have contacted the state NMEP representative.  

 Thank you for your time and suggestions.

# Reviewer report: Reviewer response for version 1

**DOI:** 10.21956/openresafrica.16984.r32323

**Published:** 2025-08-23

**Authors:** Emmanuel Akpan

**Affiliations:** 1 Federal College of Medical Laboratory Science and Technology, Jos, Nigeria

Summary

 The study viewed covering water containers as an effective preventive measure to reduce asymptomatic malaria towards the end of the rainy season. It aimed to systematically examine the prevalence of asymptomatic malaria at this time and to identify sociodemographic factors influencing malaria infections in communities within Nnewi North LGA. The findings showed that malaria transmission in Anambra State continues, peaking during the rainy season, with a 26.15% rate of asymptomatic malaria among adults in Nnewi North LGA, three times higher than among children under five. The rate of asymptomatic malaria was unaffected by socioeconomic or demographic factors, primarily due to partial immunity in adults. Despite widespread awareness of malaria and preventive tools like ITNs, many participants depended on personal assumptions for diagnosis, exposing a gap in accurate detection and treatment. Notably, covering water containers (a form of larval source management) proved to be an effective preventative measure. While herbal remedies were also used and associated with lower asymptomatic rates, their interaction with standard antimalarial treatments like ACTs remains uncertain and potentially risky. The results highlight the importance of targeted health education, improved environmental sanitation, and stronger community and political involvement to eliminate malaria effectively.

 Strengths

 This study provides valuable insights into larval source management by emphasizing the effectiveness of covering water containers as a preventive measure against asymptomatic malaria. This simple, low-cost intervention offers a promising approach for reducing malaria transmission, especially during periods of low transmission, such as the end of the rainy season. The study significantly contributes to the body of evidence supporting environmental control methods for malaria. Additionally, the conclusions are reinforced by a high response rate and well-analyzed data that add credibility to the findings.

 Weaknesses

 The use of convenience sampling and non-random recruitment introduces selection bias, limiting the generalizability of the findings. Potential participants who were excluded due to time constraints or a fear of needles may have differed in significant ways, such as healthcare behavior or malaria exposure risk, thereby skewing the sample toward individuals who are more health-conscious or accessible.

 Areas for Improvement

 Future research should focus on improving sampling methods by using randomized or stratified sampling techniques to reduce bias and obtain a more representative sample. Additionally, including home-based recruitment and data collection strategies would help reach individuals who do not often visit public spaces, thereby enhancing inclusivity and external validity.

 Minor Correction

 Line 11, under ‘Discussion’, ‘the malaria parasite’, is repeated.

Is the work clearly and accurately presented and does it cite the current literature?

Yes

If applicable, is the statistical analysis and its interpretation appropriate?

Yes

Are all the source data underlying the results available to ensure full reproducibility?

Yes

Is the study design appropriate and is the work technically sound?

Yes

Are the conclusions drawn adequately supported by the results?

Yes

Are sufficient details of methods and analysis provided to allow replication by others?

Yes

Reviewer Expertise:

Biostatistics; Infectious Disease Modeling

I confirm that I have read this submission and believe that I have an appropriate level of expertise to confirm that it is of an acceptable scientific standard.

# Response

**Published:** 2025-10-07

**Authors:** Linda Onyeka Anagu

Dear Emmanuel Akpan,

 Thank you for your input and time spent reviewing my manuscript.

# Reviewer report: Reviewer response for version 1

**DOI:** 10.21956/openresafrica.16984.r32320

**Published:** 2025-08-23

**Authors:** Solomon Oyeleye

**Affiliations:** 1 Caleb University, Imota, Nigeria

This is a timely intervention in the area of malaria prevention, especially among low income communities. The study indicates how a preventive measure of covering water containers can help to reduce asymptomatic malaria particularly towards the end of the rainy season in a South-eastern community in Nigeria. The  issue of assumption by respondents as a source of knowing malaria is a good contribution to the literature because assumption has the likelihood of promoting self medication and can lead to prevalence of drug resistant malaria.

 However, if community members only cover their containers and neglect drains and other elements outside that can serve as breeding ground for mosquitoes, that would mean exposure to mosquito bite remain unchecked. 

 Overall, while this work is commendable, it is hard to accept it as Observational study as indicated by the authors. Also, there is need to update the literature on under-5 data. Instead of using the 2018 NDHS, the authors could leverage on the availability of the updated NDHS 2025.

Is the work clearly and accurately presented and does it cite the current literature?

Yes

If applicable, is the statistical analysis and its interpretation appropriate?

I cannot comment. A qualified statistician is required.

Are all the source data underlying the results available to ensure full reproducibility?

Yes

Is the study design appropriate and is the work technically sound?

Yes

Are the conclusions drawn adequately supported by the results?

Yes

Are sufficient details of methods and analysis provided to allow replication by others?

Yes

Reviewer Expertise:

Health Communication

I confirm that I have read this submission and believe that I have an appropriate level of expertise to confirm that it is of an acceptable scientific standard.

# Response

**Published:** 2025-10-07

**Authors:** Linda Onyeka Anagu

Dear Solomon Oyeleye,

 Thank you for the time that you spent reviewing my manuscript.

 The study area section has been modified to reflect the importance of water storage containers. This study is observational as we did not intervene; we only measured some indices. NDHS 2025 is ongoing, and so there is no available data yet from this survey.

# Reviewer report: Reviewer response for version 1

**DOI:** 10.21956/openresafrica.16984.r32325

**Published:** 2025-08-19

**Authors:** Le Minh Dat

**Affiliations:** 1 Hanoi Medical University, Hanoi, Vietnam

Abstract

 Excellent — the abstract captures and accurately reflects the main content of the paper

 1. Introduction

 • Effective, clear, and well-organized

 • Introduces and sets the next section in context

 • Explains the significance of the study

 But 2 sentences left me quite confused

 - Why did you mention this information: "Anambra has the second lowest malaria incidence among under-5s residing in Nigeria", which seems irrelevant to the study

 - "Malaria incidence continues to decline in Anambra State", specifically, by how much % over how long, compared to neighboring countries, more or less

 2. Methods

 The study design was well-conceived and effectively addressed the research question.

 Participants were described comprehensively, with conditions clearly defined, as well as clear inclusion and exclusion criteria.

 The methodology is well detailed, and the main outcomes are clearly defined and appropriately measured.

 The study demonstrates ethical rigor, with approval from the ethics committee or Institutional Review Board (IRB).

 Previously published methods are properly cited, ensuring transparency and reproducibility.

 The study questionnaire was standardized and thoroughly tested before implementation, which enhances reliability and validity.

 I have two additional comments for your consideration:

 - Although you have clearly stated the name of the RDT used in the study, I suggest that you provide more details about its sensitivity and specificity, as these parameters have important implications for the accuracy of identifying positive cases.

 - Regarding the sampling method, you describe it as a “stratified convenience sampling technique”. However, based on your description, it seems more consistent with purposive sampling. I recommend reviewing and clarifying the terminology used to ensure methodological accuracy.

 3. Results:

 Results are both relevant and reliable, providing clear answers to the question.

 - In table 2, I think that the history of malaria is not knowledge about malaria, but it is just a factor related to knowledge. Knowledge about malaria includes: understanding of symptoms, agents, harms, prevention measures....

 4. Discussion

 clear arguments and specific evidence, the limitations of the study should be put at the end of the discussion, and additional directions for further studies should be added.

 Final comments: This is a good study with a lot of data and specific evidence that not everyone can access. I have read all the attached appendices, in the future, the group can do a retrospective study to evaluate the overall change in the number of malaria cases in the locality when applying intervention measures.

Is the work clearly and accurately presented and does it cite the current literature?

Yes

If applicable, is the statistical analysis and its interpretation appropriate?

Yes

Are all the source data underlying the results available to ensure full reproducibility?

Yes

Is the study design appropriate and is the work technically sound?

Yes

Are the conclusions drawn adequately supported by the results?

Yes

Are sufficient details of methods and analysis provided to allow replication by others?

Yes

Reviewer Expertise:

malaria, public health

I confirm that I have read this submission and believe that I have an appropriate level of expertise to confirm that it is of an acceptable scientific standard.

# Response

**Published:** 2025-08-21

**Authors:** Linda Onyeka Anagu

Dear Le Minh Dat,

 Thank you for your engaging and comprehensive review.

 We will include additional information and provide clarifications where necessary, as suggested.

 Thank you also for the suggestions for our future study.

# Reviewer report: Reviewer response for version 1

**DOI:** 10.21956/openresafrica.16984.r32276

**Published:** 2025-07-24

**Authors:** Johanna P Daily

**Affiliations:** 1 Albert Einstein College of Medicine, New York, USA

This study tests the carriage rate of adults in a region of Nigeria and conducts survey to assess participant knowledge of malaria. They studied 130 healthy adults. Result includes 26% RDT positive.  A survey questions regarding cost of malaria prevention tools; they report that malaria positivity was lower in those who said that the costs is reasonable compared to those who though the cost was unreasonable. 

 It is important to identify asymptomatic carriers to inform malaria control programs. Understanding the community perception on what malaria is, if they are affected and how they try to prevent malaria is helpful. 

 1. the sample size is very small to have meaningful differences in the various analyses they perform, it may be sufficient to determine the positive rate, but i am not convinced that it allows sufficient power to then test additional associations. 

 2. RDTs are much less sensitive than nucleic acid tests to detect asymptomatic parasitemia, the positivity rate is likely much higher, this is a caveat to the results. 

 3. The title states that covering water is protective from malaria I did not see that result in the paper (may have missed it)

 4. They state that those who agreed that the cost of malaria prevention was acceptable had lower malaria rates, yet the CI crosses 1, which means it is not statistically significant.

 5. I am surprised that they did not ask the participants if fever or chills was a sign of malaria, this is more specific than things like personal feelings/assumptions (i.e. not sure what that means)

Is the work clearly and accurately presented and does it cite the current literature?

Yes

If applicable, is the statistical analysis and its interpretation appropriate?

Partly

Are all the source data underlying the results available to ensure full reproducibility?

Yes

Is the study design appropriate and is the work technically sound?

Yes

Are the conclusions drawn adequately supported by the results?

Partly

Are sufficient details of methods and analysis provided to allow replication by others?

Yes

Reviewer Expertise:

malaria

I confirm that I have read this submission and believe that I have an appropriate level of expertise to confirm that it is of an acceptable scientific standard, however I have significant reservations, as outlined above.

# Response

**Published:** 2025-08-21

**Authors:** Linda Onyeka Anagu

Dear Johanna Daily,

 I am overjoyed that you were able to review this study.

 Thank you very much for your input and insight. Here are my responses.

 1. The sample size may be small for the association analysis, but we had to calculate the sample size based on our main objective - determining the positive rate.

 2. Yes, RDTs are much less sensitive than nucleic acid tests to detect asymptomatic parasitemia due to the expected low parasitemia. This is a limitation of this study, but this is what we could afford to do. 

 3. The result supporting this is in the supplement and will now be presented in the main body of the report.

 4. To double-check this, we will redo the statistics analysis on the 'agreement that the cost of malaria prevention was acceptable had lower malaria rates', and recalculate the CI. However, please note that this is a 90% CI and was mislabelled as 95%, but the p-value is listed as < 0.1.

 5. We had an item in section B of the questionnaire that covers signs and symptoms, as shown in the deposited underlying 'Consent forms and RESEARCH QUESTIONNAIRE_Malaria_Epi Study.pdf'. The actual data is not presented in the questionnaire responses either. however, this data was collected and even presented by the student at his defence. I missed this while reporting and compiling this report, and I believe it is essential to include it. This is an oversight.

 Thank you for pointing this out.   

 We will upload a second version that contains all the necessary corrections.

# Response

**Published:** 2025-09-08

**Authors:** Linda Onyeka Anagu

Dear Joana, thank you for reviewing my manuscript. It is indeed a privilege. Please see my responses below:

 1. Yes, the sample size may not be enough to test additional associations, and this is the limitation of this study, but we had to focus on our primary objective in calculating the sample size. 

 2. Yes, other nucleic acid tests would be more appropriate, but we had limited funding. 

 3. Yes, you missed the result; it was in the supplement section. The title has been equally adjusted to show that it is an association.

 4. The CI in this case crosses 1, but is still statistically significant at p < 0.1.

 5. We did ask if fever was a sign of malaria; unfortunately, this was not included while compiling the data for the manuscript. I apologize for this. It was certainly presented in the student's external examination.
